# MicrobioSee: A Web-Based Visualization Toolkit for Multi-Omics of Microbiology

**DOI:** 10.3389/fgene.2022.853612

**Published:** 2022-04-08

**Authors:** JinHui Li, Yimeng Sang, Sen Zeng, Shuming Mo, Zufan Zhang, Sheng He, Xinying Li, Guijiao Su, Jianping Liao, Chengjian Jiang

**Affiliations:** ^1^ State Key Laboratory for Conservation and Utilization of Subtropical Agro-Bioresources, Guangxi Research Center for Microbial and Enzyme Engineering Technology, College of Life Science and Technology, Guangxi University, Nanning, China; ^2^ National Engineering Research Center for Non-Food Biorefinery, State Key Laboratory of Non-Food Biomass and Enzyme Technology, Guangxi Key Laboratory of Bio-Refinery, Guangxi Research Center for Biological Science and Technology, Guangxi Academy of Sciences, Nanning, China; ^3^ Guangxi Birth Defects Prevention and Control Institute, Maternal and Child Health Hospital of Guangxi Zhuang Autonomous Region, Nanning, China; ^4^ Key Laboratory of Industrial Biotechnology, School of Biotechnology, Ministry of Education, Jiangnan University, Wuxi, China; ^5^ School of Computer and Information Engineering, Nanning Normal University, Nanning, China

**Keywords:** MicrobioSee, metagenome, integration analysis, visualization toolkit, stacked column chart

## Abstract

With the upgrade and development of the high-throughput sequencing technology, multi-omics data can be obtained at a low cost. However, mapping tools that existed for microbial multi-omics data analysis cannot satisfy the needs of data description and result in high learning costs, complex dependencies, and high fees for researchers in experimental biology fields. Therefore, developing a toolkit for multi-omics data is essential for microbiologists to save effort. In this work, we developed MicrobioSee, a real-time interactive visualization tool based on web technologies, which could visualize microbial multi-omics data. It includes 17 modules surrounding the major omics data of microorganisms such as the transcriptome, metagenome, and proteome. With MicrobioSee, methods for plotting are simplified in multi-omics studies, such as visualization of diversity, ROC, and enrichment pathways for DEGs. Subsequently, three case studies were chosen to represent the functional application of MicrobioSee. Overall, we provided a concise toolkit along with user-friendly, time-saving, cross-platform, and source-opening for researchers, especially microbiologists without coding experience. MicrobioSee is freely available at https://microbiosee.gxu.edu.cn.

## Introduction

Microorganisms are ubiquitous on earth and play a prominent role in the material cycle, climate change, and human health (Lynch and Pedersen, 2016; Crowther et al., 2019; Jansson and Hofmockel, 2020; Keohane et al., 2020). In the last 2 decades, the development of high-throughput velocimetry allowed us to observe the structure of microbial communities, in which the Earth Microbiome Project (EMP) and the Human Microbiome Project (HMP) have achieved fruitful results (Turnbaugh et al., 2007; Human Microbiome Project, 2012; Gilbert et al., 2014; Thompson et al., 2017). In the last few years, a lot of studies on the interaction between various community microorganisms and their hosts have emerged (Lundberg et al., 2012; Ren et al., 2021). Numerous studies have discovered that the loss of gut microbiota homeostasis exerts a significantly negative impact on Alzheimer’s disease, obesity, cancer, and depression. (Dao et al., 2016; Zheng et al., 2016; Flemer et al., 2017; Kim et al., 2020; Morais et al., 2021). In addition to the metagenomics technique, the multi-omics technique has been used to explore specific interactions between microorganisms and hosts in a greater depth (Lloyd-Price et al., 2019). The Integrative Human Microbiome Project (iHMP) has conducted a series of integration studies to explore human microbial–host interactions from the multi-omics data in three physiological or pathological states of human prematurity, inflammatory bowel disease, and prediabetes (Integrative, 2019).

In multi-omics studies, there are two major challenges for researchers. One is that data integration and analysis consume considerable time and effort of researchers because of the complex usage of combining tools (Lin et al., 2020), and the other is that the existing drawing tools cannot satisfy the demands for describing high-dimensional data, which poses stress for the researchers to represent the analysis results of microbiome data (Sinha et al., 2015; Ramirez et al., 2018). Visualizing the results of data obtained from multi-omics studies is a huge burden for the researchers (Lin et al., 2020).

Currently, various tools have emerged and were used to analyze multi-omics data and visualize omics results. The main steps of the metagenome analysis are clustering or denoising the raw data to obtain abundance tables. In the analysis phase, QIIME2 (Hall and Beiko, 2018; Knight et al., 2018; Rai et al., 2021) performs better than other tools (Straub et al., 2020). However, users have to use dedicated tools to view graph files generated from QIIME2, which adds burden for the users to observe the results (Min et al., 2021).

For big data obtained from the multi-omics studies, visualizing the analysis results requires easier tools (Chen et al., 2020). Traditional graphing tools exhibited excellent graphing capabilities and convenience, especially client tools that are user-friendly but have problems such as low system compatibility and high costs. For example, OriginPro and GraphPad Prism v9 are two drawing tools for the general public, with rich styles of charts but of high economic costs. Command line-based tools, such as the Matplotlib package in Python and the ggplot2 package in R, provide novel graphing methods and graph styles with excellent plotting capabilities and are usually used for the secondary development (Skidmore et al., 2016; Wagih, 2017; Tareen and Kinney, 2020; Liu et al., 2021; Xu et al., 2021). A lot of command line-based tools which are cross-systems and open sources have been developed by bioinformaticians to exhibit the results of the multi-omics data (Ito and Murphy, 2013). However, those command line-based tools require users to spend considerable time learning a programming language, which decreases the efficiency for scientific researchers in non-computer fields, especially doctors and experimenters.

**FIGURE 1:**
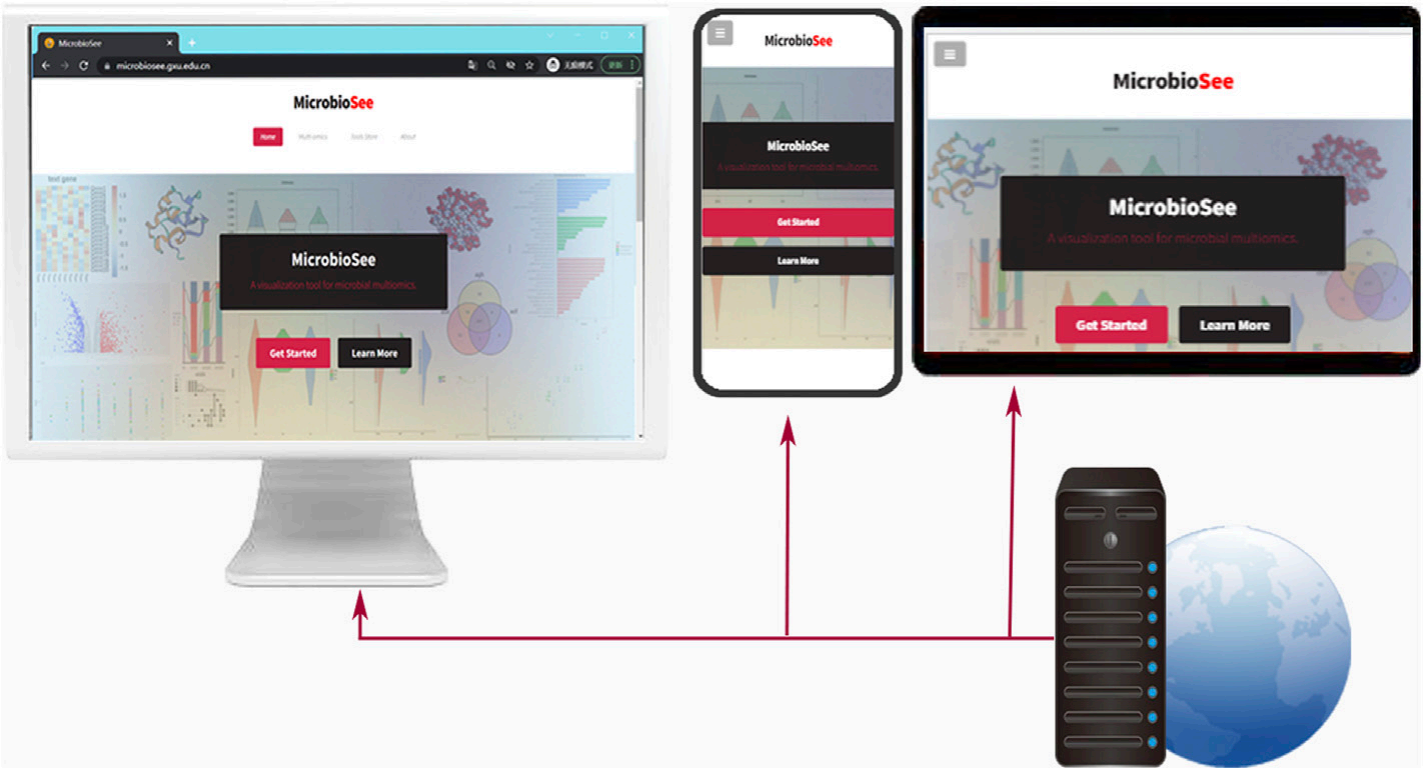
Presentation of index pages of MicrobioSee on different devices, including PC, Pad, and smartphone. The capabilities of the cross-platform are built by a webserver. The index page was designed with responsive web layouts, which enables users to get the best visual effects on different devices.

For these issues, we developed MicrobioSee, a web-based toolkit for multi-omics visualization, which is a cross-platform, user-friendly, time-saving, and an open source (Figure 1). The idea of this tool originated from the fact that conventional charts and tools cannot satisfy the demands of visualization for researchers without programming experience in their multi-omics studies. MicrobioSee is an efficient toolkit for visualization that eliminates high costs for users without programming experience.

## Methods

**FIGURE 2:**
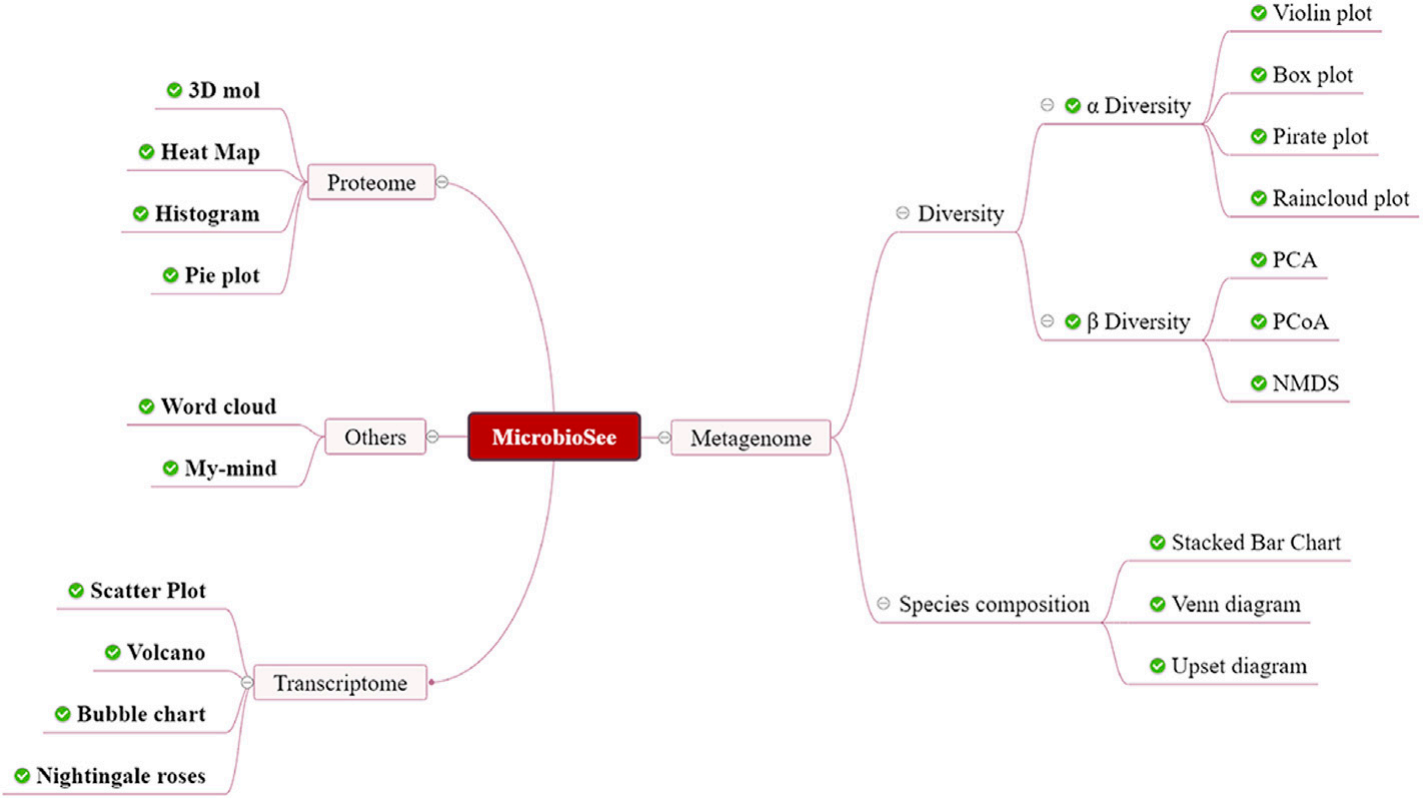
Structure of MicrobioSee. MicrobioSee comprises four modules: metagenome, proteome, transcriptome, and others. In total, 20 graph styles could be plotted into by MicrobioSee so far.

**FIGURE 3:**
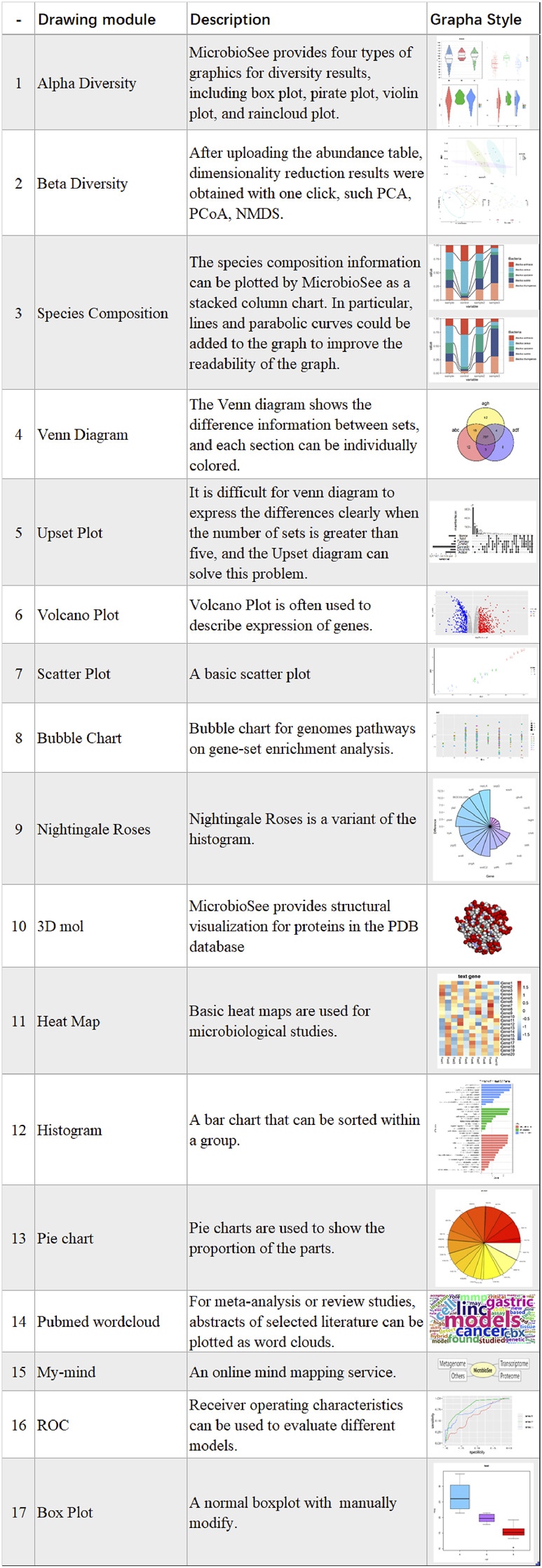
Seventeen drawing modules and related introduction.

MicrobioSee was developed for microbiome multi-omics data, such as the metagenome, proteome, and transcriptome (Figure 2). The whole website was divided into front-end and back-end. In the front-end, the VUE technology is used to render the interface. In the back-end, the R program was used for responding to users’ interactions and built-in drawing commands. In addition, most of the tools in MicrobioSee were built with shiny services to achieve real-time interactive plotting. A series of graphics could be plotted interactively by MicrobioSee, including the rose plot, heat map, box plot, upset plot, Venn diagram, and so on, which could be applied to multi-omics studies (Figure 3). The options for height, width, and resolution in each drawing module were provided for plotting.

### Main Function

Alpha diversity that describes the microbial community composition is a critical index of the metagenome (Walters and Martiny, 2020). The results of alpha diversity from the USEARCH program or vegan package (Oksanen et al., 2013) could be plotted into four graph styles by MicrobioSee, including the box plot, raincloud plot, and violin plot. The pirate plot (Phillips, 2017) and the raincloud plot (Allen et al., 2019), composed of various graph styles, are more intuitive than others in aesthetics. The analysis of variance (ANOVA) and Kruskal–Wallis test were used to determine the difference between the groups for alpha diversity. The nonparametric statistical tests were realized by the aov function and the Kruskal–Wallis test function in R. After clicking the start button, users can obtain the selected graph style and test.

Beta diversity is used to describe the variability in species composition between the groups (Whittaker, 1960). Plenty of indicators are used to measure beta diversity, and the Bray–Curtis similarity index is the main one (Bray and Curtis, 1957; Tuomisto, 2010). The distance matrix, generated from operational taxonomic units (OTUs), abundance table, or exact sequence variant tables, is calculated by the vegan package in MicrobioSee. After the dimensionality reduction, results would be plotted with the vegan package. There are three methods for the dimensionality reduction of the distance, including principal component analysis (PCA), principal coordinates analysis (PCoA), and non-metric multidimensional scaling (NMDS). Analysis of similarities (ANOSIM) was used to determine the similarity among the groups in the PCoA plots from MicrobioSee.

The species composition of the microbial community, one of the most cardinal factors to determine the nature of the community, is the basic characteristic to identify the different community types (Bell et al., 2005; Burrows et al., 2019; Jones et al., 2021). The structure of the species composition is usually plotted into a basic stacked column chart, but it cannot visually represent small differences for the adjacent groups. In MicrobioSee, lines could be added to the graphs among the numerical points of the adjacent groups in a stacked column chart. In addition, the curves could be added to the stacked column charts in a way that parabolic functions are generated by the relations between the taxon in the adjacent groups, which makes the stacked column charts intuitive and elegant. Each parabola would be calculated from the vertex of units and the midpoint of the adjacent units. Although the lines or curves added do not contain any scientific meaning, it could be valuable for users to visualize their results as a stacked column chart with lines or curves.

The screening literature is usually performed at the beginning of integrating data for target subjects. The metadata information of the selected literature, such as titles, abstracts, and keywords, could be accessed and downloaded easily by the crawler technology in MicrobioSee. The metadata would be automatically plotted into word clouds by the ggplot2 package, which describes the characteristics of the integrated literature. MicrobioSee could help researchers identify hot methods of research relating to target keywords.

The metagenome integrated data belong to the big data, and building models for classification and prediction is the most popular application in big data techniques, such as machine learning techniques (Cammarota et al., 2020; Namkung, 2020). For binary classification models, such as in sickness and in health, the receiver operating characteristic (ROC) curves are usually used to evaluate the quality of the models. The area under the curve (AUC), an important feature of the ROC, is one of the most commonly used metrics (Wang and Guo, 2020). The abundance tables of multiple taxonomic levels and metadata were used for constructing models by machine learning software (Yuan et al., 2020). In MicrobioSee, multiple ROC curves from various models were rendered by the pROC package (Robin et al., 2011) in R, which could identify the qualities for the better models. According to the specificity and sensitivity in graphs generated from MicrobioSee, users could evaluate and choose the models of various species classification levels or model methods.

## Case Studies

To display the utility of MicrobioSee, three case studies were chosen and visualized by MicrobioSee. For brevity, we cannot explore all the functions of MicrobioSee but focus on the visualization of the most common scientific questions. Relevant data in case studies can be made available in the supplementary files.

### Case 1

**FIGURE 4:**
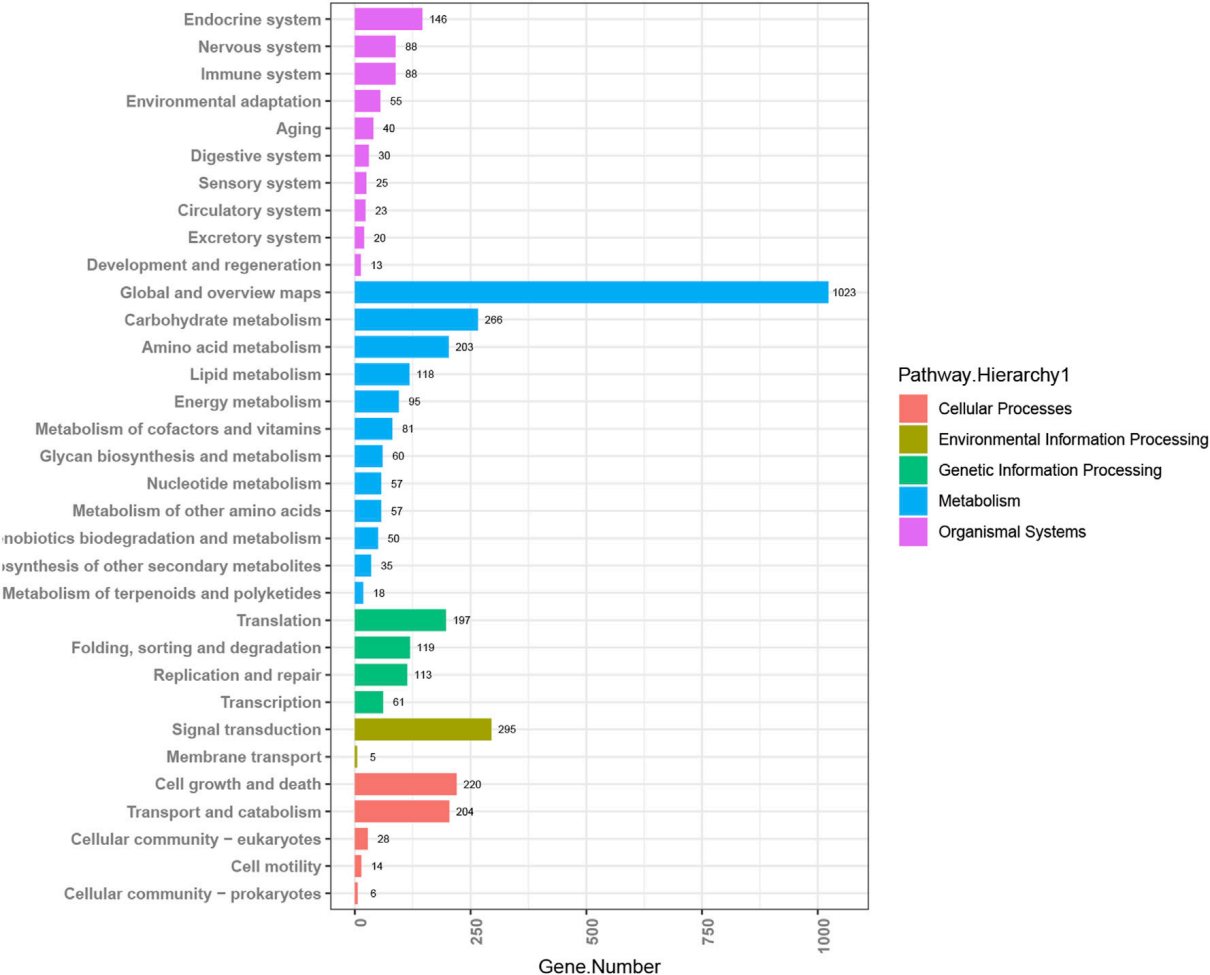
Visualization of KEGG annotations from case study 1 by MicrobioSee. The data in the enrichment pathway of the differentially expressed genes (DEGs) among the groups were plotted into a histogram with an order in each group by MicrobioSee.

By MicrobioSee, users can plot a histogram easily with axis transposition and group sorting with one click. The data for this example are from the study of copper tolerance in *Meyerozyma guilliermondii* GXDK6, which was screened from the mangrove sediments (Bu et al., 2021). Multi-omics techniques were used to explore the tolerance mechanisms of the target strain with different copper ion concentrations. After annotating with the KEGG database, the enrichment pathways of differentially expressed genes (DEGs) from the GXDK6 transcriptomics analysis at 600 ppm copper concentration were plotted into a histogram with an order by MicrobioSee (Figure 4). The histogram generated by MicrobioSee can be sorted within the groups, which is more intuitive for users to observe the ranking of the annotation.

### Case 2

**FIGURE 5:**
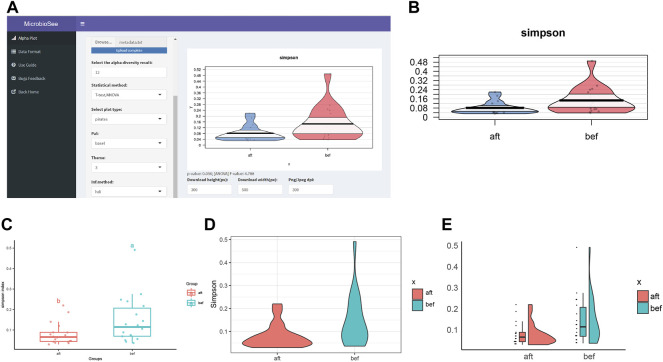
Process and visualization of the diversity analysis from case study 2 by MicrobioSee. **(A)** Panel of the alpha plot with a pirate plot in MicrobioSee. **(B)** Pirate plot was generated from case study 2 by MicrobioSee. **(C)** Box plot was generated from case study 2 by MicrobioSee. **(D)** Violin plot was generated from case study 2 by MicrobioSee. **(E)** Raincloud plot was generated from case study 2 by MicrobioSee.

The samples were selected from the study of fecal microbiota transplantation (FMT) (Zhang et al., 2021). The study successfully collected 16S sequencing data of 18 constipated patients before and after FMT. Based on the raw data and analysis methods provided in the literature, we obtained the diversity results and plotted the Simpson index into the four graph styles by MicrobioSee (Figure 5). According to the statistical analysis from MicrobioSee, the Simpson index increased significantly after FMT.

### Case 3

**FIGURE 6:**
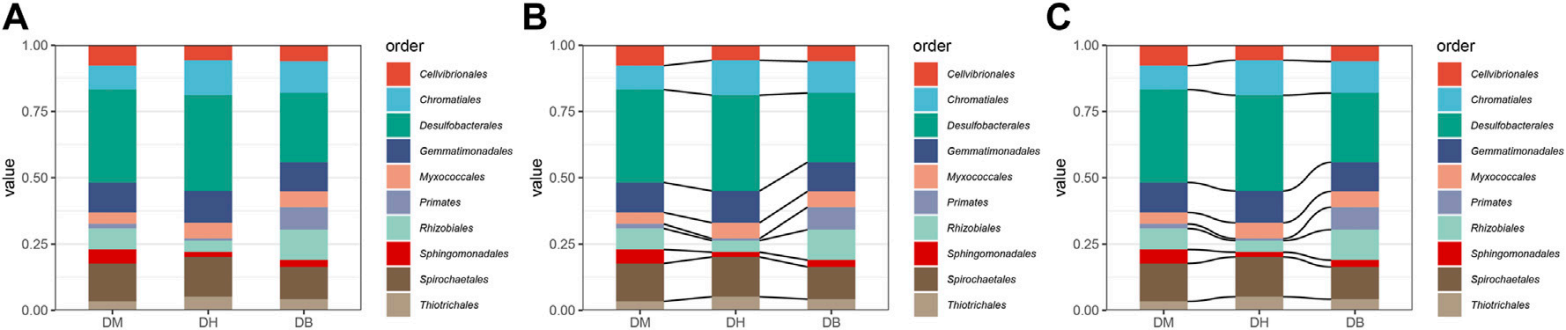
Relative abundance of the top 10 species in case study 3 was plotted with the three stacked column charts by MicrobioSee. **(A)** Stacked column chart without lines. **(B)** Stacked column chart with straight lines. **(C)** Stacked column chart with curves.

Parabolic curves or straight lines were added to the stacked column charts, and the differences in the relative abundance of the species among groups would be observed clearly. The sample chosen was collected from the National Shankou Natural Reserve of Mangrove in the Beibu Gulf of China (Nie et al., 2021). The relative abundance of the top 10 orders in the dry season was plotted into the stacked column charts with lines and curves by MicrobioSee (Figure 6). In the M and H regions, the relative abundance of Desulfobacterales is similar and greater than B in the dry season.

## Results and Discussion

In this work, we developed MicrobioSee, a web-based toolkit for the multi-omics studies, which contains plenty of computer technologies. The burden of plotting the result of the multi-omics studies would be eased with MicrobioSee. The pirate plot and raincloud plot are utilized as complements to the existing graph styles for visualization of alpha diversity results. Benefiting from the abundant R package resources, 17 plotting modules were developed for MicrobioSee. MicrobioSee also offers a few innovations in aesthetics. For example, elegant parabolic curves were added to the stacked column charts which would make them more aesthetically pleasing in the visualization of species composition.

The functions and advantages of the eight tools were summarized (Table 1). The vegan package (Oksanen et al., 2013) and phyloseq package (McMurdie and Holmes, 2013) are recognized by many researchers, but it is not friendly for researchers because of lacking interfaces. In the studies of the amplicon, QIIME2 (Hall and Beiko, 2018) and USEARCH (Edgar, 2010) are popular for high-speed analysis, but it is rarely used in the visualization of results on account of the insufficient number of their graphic styles. TBtools (Chen et al., 2020) has been popular with Windows users since it was developed, but it is disappointing for users of other platforms. Web-based tools could be used directly through a modern browser without platform limits. Animalcules (Zhao et al., 2021) provides an interface by Shiny technology, but it is a localized service with complex dependencies and could be installed with errors. Metaviz (Wagner et al., 2018) provides a web application for interactive visualization of the microbiome, but the methods of metaviz are not comprehensive or specific for 16S rRNA, metagenomic, or transcriptomic data. MicrobiomeAnalyst (Dhariwal et al., 2017; Chong et al., 2020), an excellent web toolkit in the field of downstream microbiome analysis, provides analysis and visualization, but few visual graphic styles are available. MicrobioSee was developed for interactive visualization of the microbiome, and microbiologists could use it for free and install it for free after short learning. The results in various omics studies can be visualized by MicrobioSee besides 16S rRNA and shotgun sequencing microbiome data. Due to its flexibility, it can also be applied in other fields. As long as uploading is in the same format as the sample data, the images of the corresponding graphic style would be obtained.

**TABLE 1:** Comparisons of MicrobioSee and other popular visualization tools.

	Vegan	USEARCH11 64-bit	Phyloseq	Metaviz	Qiime2	OriginPro	GraphPad Prism v9	Animalcules	TBtools	MicrobiomeAnalyst	MicrobioSee
Literature integration											✔
3D molecular structure											✔
Installation-free										✔	✔
Interface				✔	✔	✔	✔	✔	✔	✔	✔
Interactive visualization			✔	✔	✔	✔	✔	✔	✔	✔	✔
Diversity visualization	✔	✔	✔	✔	✔	✔	✔	✔		✔	✔
Free use	✔		✔	✔	✔			✔	✔	✔	✔
Language/platform	R	Linux	R	R	Linux	Windows	Windows/macOS	R	Windows	Modern browsers	Modern browsers

The features are assessed using the symbol “✔” for “present.”

For a more convenient operation, the tool would be continuously updated. Compared with client tools, it is unnecessary to be reinstalled when a new version is released, which is user-friendly. Inconveniently, web tools are highly dependent on the web environment (Chen et al., 2020) and so is MicrobioSee. When the number of users increases to a certain extent, the servers and bandwidth for MicrobioSee need to be expanded. The servers of MicrobioSee may suffer from attacking for the global open access, and the firewalls need to be constantly upgraded.

## Conclusion

In total, 17 plotting modules have been built for MicrobioSee so far, such as the violin plot, box plot, rose plot, heat map, box plot, upset plot, and Venn diagram. Although most functions are not unique to MicrobioSee, they were combined, optimized, and interfaced for researchers with limited coding experience. MicrobioSee simplifies the methods for users without programming experience.

## Data Availability

The original contributions presented in the study are included in the article/Supplementary Material, further inquiries can be directed to the corresponding authors.
